# Wound Edge Protectors in Open Abdominal Surgery to Reduce Surgical Site Infections: A Systematic Review and Meta-Analysis

**DOI:** 10.1371/journal.pone.0121187

**Published:** 2015-03-27

**Authors:** André L. Mihaljevic, Tara C. Müller, Victoria Kehl, Helmut Friess, Jörg Kleeff

**Affiliations:** 1 Department of Surgery, Klinikum rechts der Isar, Technische Universität München, Ismaninger Strasse 22, 81675 Munich, Germany; 2 Institute for Medical Statistics and Epidemiology, Klinikum rechts der Isar, Technische Universität München, Ismaninger Strasse 22, 81675 Munich, Germany; University Hospital Oldenburg, GERMANY

## Abstract

**Importance:**

Surgical site infections remain one of the most frequent complications following abdominal surgery and cause substantial costs, morbidity and mortality.

**Objective:**

To assess the effectiveness of wound edge protectors in open abdominal surgery in reducing surgical site infections.

**Evidence Review:**

A systematic literature search was conducted according to a prespecified review protocol in a variety of data-bases combined with hand-searches for randomized controlled trials on wound edge protectors in patients undergoing laparotomy. A qualitative and quantitative analysis of included trials was conducted.

**Findings:**

We identified 16 randomized controlled trials including 3695 patients investigating wound edge protectors published between 1972 and 2014. Critical appraisal uncovered a number of methodological flaws, predominantly in the older trials. Wound edge protectors significantly reduced the rate of surgical site infections (risk ratio 0.65; 95%CI, 0.51–0.83; p = 0.0007; I^2^ = 52%). The results were robust in a number of sensitivity analyses. A similar effect size was found in the subgroup of patients undergoing colorectal surgery (risk ratio 0.65; 95%CI, 0.44–0.97; p = 0.04; I^2^ = 56%). Of the two common types of wound protectors double ring devices were found to exhibit a greater protective effect (risk ratio 0.29; 95%CI, 0.15–0.55) than single-ring devices (risk ratio 0.71; 95%CI, 0.54–0.92), but this might largely be due to the lower quality of available data for double-ring devices. Exploratory subgroup analyses for the degree of contamination showed a larger protective effect in contaminated cases (0.44; 95%CI, 0.28–0.67; p = 0.0002, I^2^ = 23%) than in clean-contaminated surgeries (0.72, 95%CI, 0.57–0.91; p = 0.005; I^2^ = 46%) and a strong effect on the reduction of superficial surgical site infections (risk ratio 0.45; 95%CI, 0.24–0.82; p = 0.001; I^2^ = 72%).

**Conclusions and Relevance:**

Wound edge protectors significantly reduce the rate of surgical site infections in open abdominal surgery. Further trials are needed to explore their effectiveness in different risk constellations.

## Introduction

An estimated 234,2 million surgeries are performed annually resulting in more than 7 million complications worldwide [1]. Of these surgical site infections (SSI) are one of the most frequent complications following abdominal surgery and cause considerable morbidity, mortality and health-care costs. Despite the implementation of preventive measures SSI rates in prospective trials with adequate follow-up and standardized SSI definition in abdominal surgical patients remain high and vary from 15% to 30% [2–5]. In the U.S. an estimated 300,000 to 500,000 SSIs occur annually [6–8]. SSIs are associated with a twofold increased relative risk of in-hospital mortality [8] and over one third of postoperative deaths in patients with SSIs are attributable to the infection [9]. Furthermore, several studies have shown an increase in the length of hospital stay between 6–24 days [8,10–13]. The resulting direct costs have to be added to the indirect costs resulting in substantial expenses to the health care system and the society.[14–16].

The incidence of postoperative SSIs varies substantially depending on the degree of intraoperative contamination (clean, clean-contaminated, contaminated or dirty operations as defined by the Centers for Disease Control and Prevention, CDC [17]) and the site of operation [18–20]. Colorectal surgery has been associated with high rates of SSIs in studies with adequate design and follow-up [5,21,22]. While in the past definitions of SSIs varied, over the last decade the SSI definition of the CDC has gained universal acceptance [17].

As the most frequent pathogens causing postoperative SSIs following abdominal surgery are endogenous pathogens from the skin or gastrointestinal tract [17,23] it has been proposed that protecting the wound edges from bacterial invasion during surgery might reduce SSIs [24]. To this end impervious circular wound edge protectors (CWEP) have been proposed as in abdominal surgery as a simple, easy-to use and cost-effective intervention to reduce SSI. There are different devices available, but they all fall into two main categories: a.) devices with a single semi-rigid plastic ring placed into the abdominal cavity after laparotomy with an impervious drape attached, which comes out of the abdomen to protect the incisional edges (single-ring device) (S1 Fig); b.) devices with two semi-rigid plastic rings connected by an impervious drape. By placing one of the rings inside the abdomen after laparotomy and one externally, the drape protects the wound edges during surgery (double-ring device) (S1 Fig). While CWEPs effectively protect wound edges from bacterial invasion [25] their clinical effectiveness has been disputed and their widespread use has been hampered by conflicting trial results with some authors reporting beneficial effects [26–28] while others found no benefit [29–31]. Two previous smaller reviews based on limited patient numbers and low-quality evidence reported a beneficial effect of CWEPs, but called for adequately powered, high-quality trials [32,33] which have been conducted since then. The objective of our meta-analysis, therefore, is to evaluate the effectiveness of CWEPs in reducing SSIs compared to non-CWEP usage in patients undergoing open abdominal surgery based on all available RCTs and in a number of risk constellations.

## Methods

The review is reported in line with current PRISMA guidelines [34] (S1 PRISMA Checklist). The systematic review and meta-analysis was conducted according to a pre-specified protocol, which is available upon request.

### Eligibility Criteria

We included all RCTs in human subjects of any age comparing the use of an intraoperative CWEP with standard care in open abdominal surgery (elective and emergent). Trials were included if CWEPs were the only difference between trial arms. The CWEP intervention was defined as any intervention that covered the wound edges (i.e. skin, subcutaneous tissue, muscle and fascia) during surgery with an impervious plastic drape. We included trials using single-ring as well as double-ring devices (S1 Fig). We excluded trials evaluating adhesive incision drapes that are placed on the skin and cut during laparotomy as they do not cover the incisional edges during surgery. We included any trial that reported SSI based on clinical signs, but excluded trials that used purely microbiological outcome definitions as purely bacteriological outcome definitions inadequately differentiate between bacteriological contamination and true SSI. Furthermore, we considered SSIs at the laparotomy site only, but not at alternative sites (e.g. drainage sites which were not covered by the CWEP). No restrictions were defined for year of publication, language or length of follow-up.

### Literature search

The following databases were searched: a.) MEDLINE (1946 till April 1 week 2014) via OvidSP last search on 15^th^ April 2014; b.) MEDLINE in-process and other non-indexed citations (April 15, 2014) via OvidSP, last search on 16^th^ April 2014; c.) Cochrane Central Register of Controlled Trials (CENTRAL) (January 2014) via OvidSP, last search on 16^th^ April 2014; d.) Excerpta Medica Database (EMBASE) (1947 till April 2014, EM74) via *Deutsches Institut für Medizinische Dokumentation und Information* (DIMDI), last search on 17^th^ April 2014; e.) Web of Science Core Collection (1945 till present) via Web of Knowledge, last search 17^th^ April 2014.

**Fig 1:**
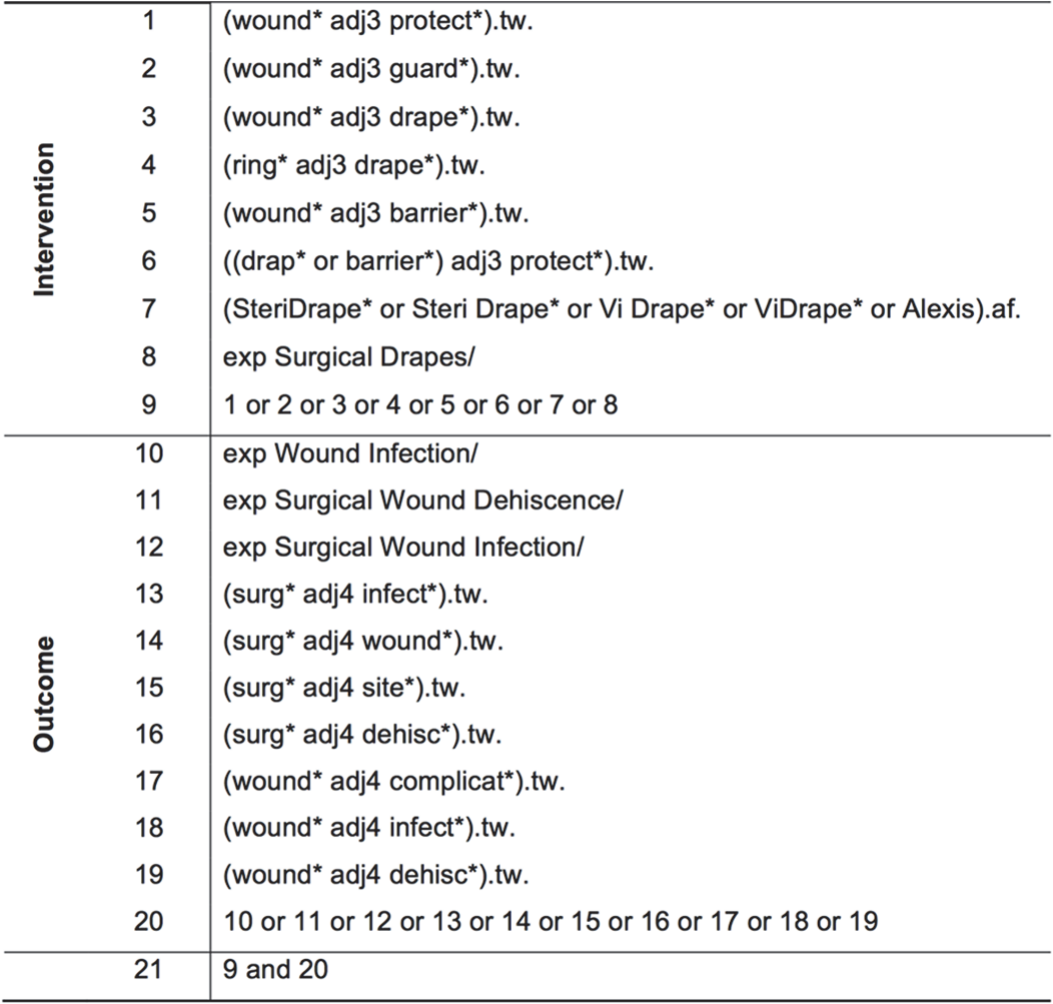
Search strategy for MEDLINE via OvidSP.

Moreover, the references of the included articles were hand-searched to identify additional relevant studies. We used the patient-intervention-comparison-outcome (PICO) scheme to build our search strategy using search terms describing the intervention (CWEP) and the outcome (SSI). Sensitive search strategies were developed for all databases using wildcards and adjacency terms were appropriate. The search strategy for the MEDLINE search via OvidSP is shown in Fig 1. The search strategies for the other databases were adapted to the specific vocabulary of each database. We combined our search of the MEDLINE database via Ovid with the Cochrane Highly Sensitive Search Strategy for identifying RCTs in MEDLINE, sensitivity maximizing version, Ovid format (2008 revision) [35].

### Trial Selection and data extraction

Titles and abstracts of studies were screened by two reviewers (A.L.M., T.C.M.) independently. In case of disagreement, a consensual decision between the two reviewers under involvement of a third independent reviewer (J.K.) was reached. Data was entered in RevMan 5 software 5.2. [36]. The following data items were extracted: year of publication, country of origin, study design, number of participants (total and for each group), type of surgery performed, risk factors for SSIs, grade of surgical contamination, inclusion and exclusion criteria, retractor type (single-ring vs. double-ring), description of the intervention, description of the control intervention, described and reported outcomes, SSI definition used, duration of follow-up, outcome data and effect sizes, number of patients lost to follow-up/withdrawals, funding and competing interests.

### Assessment of risk of bias in individual studies

Bias was judged using The Cochrane Collaboration tool of for assessing quality and risk of bias [37]. Trials were defined as having an overall high risk of bias, if they were assessed high risk in any of the following domains: random sequence generation, allocation concealment, blinding of outcome assessors or if the duration of follow-up was inadequate (less than 30 days). As blinding of surgeons performing the CWEP intervention is impossible, double-blinding is defined as blinding of patients and SSI outcome-assessors.

### Statistical methods

Risk ratio (RR) was used as summary measure for the dichotomous outcome: presence or absence of SSI. Results are presented with 95% confidence interval (CI). RevMan 5 software (version 5.2.) was used for data analysis [36]. Clinical heterogeneity was assessed. Data analysis was pre-specified using a random effects model given the variation in type of surgery and SSI definition (clinical heterogeneity). However, since random effects models do not give reliable estimates when few studies are included [38] and give more weight to small trials at the expense of large trials [39], analyses were also performed using a fixed-effects model. Statistical heterogeneity was assessed by using I^2^ statistics and results of over 60% were considered as substantial heterogeneity.

Missing data (e.g. death, re-laparotomy, lost to follow-up) were treated in the following pre-specified way: when trials specified missing data for each treatment group, deaths and re-laparotomies were counted as SSI in both the intervention and control group, because a.) SSIs are a frequent cause of re-laparotomy and/or death, b.) death and re-laparotomy are a clinical ‘worst case’ outcome; therefore, not considering death and re-laparotomies would underestimate the risk for patients. Missing data for other reasons (e.g. lost to follow-up) were considered non-SSI cases in both groups given the overall small number of SSIs. When trials reported missing data, but did not specify the trial arm, only complete case data was used for analysis. Similarly, for trials that did not specify group numbers post-randomization and before drop-out, complete case data was used for analysis. For the pre-specified sensitivity analyses other imputation methods were applied: a.) all patients excluded post-randomization/with missing data were assumed to have developed SSIs in both groups; b.) patients excluded post-randomization were assumed to have developed SSI in the CWEP group, but not have developed SSI in the control group. Furthermore, a pre-specified sensitivity analysis after removing trials at high overall risk of bias was conducted.

The risk of publication bias was assessed by means of a funnel plot for the endpoint SSI.

### Additional analyses

In addition to the sensitivity analyses described above, the following pre-specified subgroup analyses (effectiveness of CWEP vs. non-CWEP on SSI rate) were performed: a.) in colorectal vs. non-colorectal surgery; b.) for different degrees of intraoperative contamination (clean, clean-contaminated, contaminated or dirty operations as defined by the CDC [17]); c.) for single-ring CWEPs; d.) for double-ring CWEPs. Furthermore, two post-hoc sensitivity analyses were performed: a.) including only multicenter trials; b.) excluding underpowered trials with less than 40 patients per group.

## Results

### Trial characteristics

**Fig 2:**
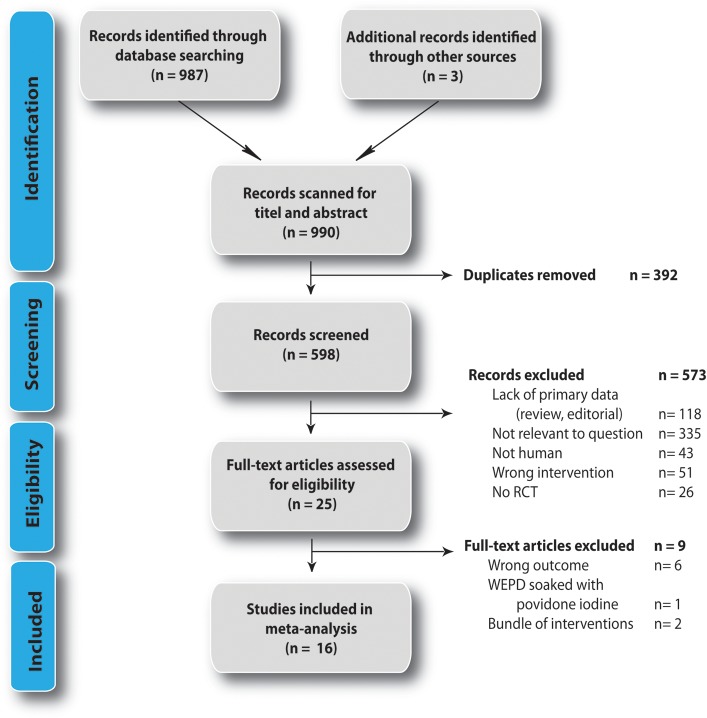
Flow diagram of studies selected according to PRISMA guidelines [34].

We identified 990 studies, 987 studies by database searching and 3 studies by hand searching. Of these 392 duplicates were removed (Fig 2). Of the remaining 598 studies titles and abstracts were assessed for eligibility and 573 studies were consequently removed according to pre-specified criteria. Of the remaining 25 trials full-text articles were retrieved, of which 9 had to be excluded on final analysis as they investigated the wrong outcome (bacteriological outcome), used a CWEP soaked in povidone-iodine (unclear if observed effect is due to CWEP, or disinfectant) or studied a bundle of interventions (S1 Table). Sixteen RCTs were included in the final meta-analysis [40–43,27,44–47,31,48,49,29,50,26,51–53].

The characteristics of the 16 included RCTs are listed in Table 1. The trials reported SSI outcomes in a total of 3695 patients, of which 1846 received CWEPs as investigational intervention. One trial investigated three patient groups [29], all other trials investigated two parallel study groups. All trials were single-center studies with the exception of the trials by Pinkney et al. (21 centers) [49], Mihaljevic et al. (16 centers) [46], Reid et al. (4 centers)[26] and Nyström, Broomé et al. (2 centres) [31]. In the reports by Sookhai [51] and Psaila [29] it remains unclear if trials were conducted at a single or two trial centers. Most trials were conducted in patients undergoing colorectal surgery [26,31,40–43] or general abdominal surgery [27–29,45,49,53]. Two trials focused specifically on patients undergoing open appendectomy[44,48] and one on women undergoing cesarean section[52]. All studies prespecified SSI as an outcome, but there was considerable variation in the definitions applied (Table 2). Six trials referred to the SSI definition of the CDC [40,42,27,45,49,26]. In the remaining studies the authors used own definitions of SSI. Furthermore, length of follow-up varied widely. Five trials did not specify the duration of follow-up [27,29,31,41,52] and 9 trials had a length of follow-up of 30 days or more as required by current guidelines [26,28,31,40,42,43,46,49,50]. Baier et al. merely conducted a telephone interview on postoperative day 30 [40].

**Table 1:** Characteristics of the included studies with description of intervention and control, risk factors for surgical site infections and level of contamination.

Study	Year	Country	Type of surgery	Inclusion criteria	Exclusion criteria	No. of centers	Retractor type	Description of intervention	Description of control	Risk factors for SSI
Baier et al.^39^	2012	Germany	Colorectal surgery	• Patients designated for laparotomy for any reason; • Elective operations	• Appendectomies • Ostomy reductions • Patients requiring a reoperation within 30 days for reasons other than SSI	1	single-ring	"We used the a 3M Steri-Drape ring drape in S,M,L according to incision length"	"wound edges were protected during surgery with wet cloth towels"	1.) all patients received antibiotic prophylaxis 2.) all patients received standard skin disinfection 3.) all patients received sterile coverage 4.) trial arms were not significantly different with respect to: wound hematoma, diverticulitis, BMI >30, ASA score >2
Bätz et al. ^40^	1987	Germany	Colorectal surgery	• Patients with colorectal carcinoma	• None specified	1	single-ring	Ring drape (Epiplast, Becton-Dickinson, Heidelberg, Germany)	Incise drape	1.) all patients received single dose prophylaxis with cephalosporins 2.) all wounds were closed primarily 3.) all patients received preoperative orthograde colonic irrigation 4.) trial arms were comparable with respect to: age, weight, T-stag 5.) type of procedure was comparable between groups
Cheng et al. ^41^	2012	Malaysia	Colorectal surgery	• Adult patients undergoing elective open colorectal surgery via a standardized • midline incision • Informed consent	• Laparoscopic approach • Contraindication for patient-controlled analgesia • Requirement for an emergency re-laparotomy	1	double-ring	"The incisions of study group patients were protected with the ALEXIS O-Ring retractor during the operation."	"Incisions of the control group were protected via a conventional method which comprised 4 abdominal packs and Balfour retraction"	1.) intravenous cefoperazone 2g and metronidazole 500mg were given for antibiotic prophylaxis 2.) incisions were standardized to 17 cm 3.) incisions were closed with absorbable subcuticular Vicryl 4.) incisions were infiltrated with 20ml of local lignocaine 1% 5.) trial arms were not significantly different with respect to procedures performed; 6.) all procedures were clean-contaminated 7.) trial arms were not significantly different with respect to: age, gender; albumin level, immune status, prior antibiotic treatment; bowel preparation duration of surgery, diabetes, length of hospital stay, ASA score
Gamble & Hopton ^42^	1984	NR (UK or Australia)	Colonic surgery	• Elective colonic surgery	• None specified	1	single-ring	"The plastic ring drape consists of a flexible, semi-rigid plasitc ring to the outer rim of which is welded a plastic sheet. The ring is compressed, inserted into the abdominal cavity and positioned under the abdominal wall inside the peritoneum. The plastic drape is smoothed out round the wound and clipped to the surrounding drapes, thus covering the edges of the incised abdominal wall, providing a barrier. . ."	"Without ring drape"	1.) bowel preparation was standardized between both groups 2.) perioperative antibiotic prophylaxis/treatment was standardized between both groups (metronidazol 200mg i.v. 8-hourly for 24 hours following surgery; ampicillin 500mg with premedication and 8-hourly for 24 hours) 3.) skin preparation was standardized between both groups (PVP-iodine and Steridrape) 4.) trial arms were comparable with respect to: gender, age, colostomies, pathology
Horiuchi et al. ^26^	2007	Japan	Nontraumatic gastrointestinal surgery	• Nontraumatic gastrointestinal surgery	• Severe adhesions with a history of laparotomy • Long-term use of steroids • Laparoscopic or minor surgery • Colon perforation	1	double-ring	"We placed the Alexis retractor in close contact with a wound margin immediately after making an incision in the abdomen."	"Wound margin was left untreated"	1.) gastrointestinal anastomotic technique was standardized between both groups 2.) standardized washing of intraperitoneal space after finishing intraabdominal procedure in both groups 3.) standardized closing of fascia and wound with wound irrigation in both groups; 4.) standardized preoperative antbiotic treatment in both groups 5.) all colorectal patients received mechanical bowel preparation using 2L of polyethylene gylcol 6.) trial arms were not significantly different with respect to: gender, age, BMI, Albumin level, length of operation, blood loss, body temperature, transfusion, maximum blood sugar level;
Lee et al. ^43^	2009	USA	Open appendectomy	• Clinical diagnosis of appendicitis • Planned open appendectomy • Informed consent	• Insulin-dependent diabetes mellitus • Inability to attend follow-up visits;	1	double-ring	"The Alexis retractor was placed in the wound upon entry into the abdominal cavity and remained in place for the duration of the procedure."	"Standard retractors"	1.) all patients received standared dose antibiotic prophylaxis; 2.) standardized wound closure for all patients; all wounds were closed primarily; 3.) antibiotic treatment was given for 24 hours for simple appendicities; until patient was afebril for 24 hours with normal white blood cell count in cases of complicated appendicities; for an additional 5–7 days in cases of ruptured appendicities 4.) trial arms were comparable with respect to: age, gender, BMI, tobacco use, diabetes, degree of appendicitis, immunosuppression;
Mihaljevic et al. ^45,46^	2014	Germany	Open abdominal surgery with median or transverse laparotomy	• Elective open abdominal surgery requiring a median or transverse laparotomy • Ability to understand extent and nature of the trial and sign written informed consent form • 18 years or older • Planned operation had to be classified as clean or clean-contaminated preoperatively according to CDC definition	• ASA grade >3 • Pregnant or lactating women • Midline or transverse laparotomy within the last 60 days prior to trial intervention • Planned relaparotomy within 30 days after trial intervention • Planned contaminated operations according to CDC definition • Small abdominal operations without planned transverse or midline laparotomy (for example, appendectomy) • Concurrent abdominal wall infections • Severe immunosuppression • Severe preoperative neutropenia (≤0.5 × 10^9^ cells/l) • Liver cirrhosis (Child-Pugh B or C)	16	single-ring	"Immediately after laparotomy patients in the intervention group received wound edge coverage (skin, subcutaneous tissue, muscle, fascia) with a single-ring CWEP (Steri-Drape Wound Edge Protector, 3M, St. Paul, MN, USA). CWEPs. . . were left in situ for the duration of the entire surgery and were removed immediately before closure of the fascia."	"Patients in the control group had their wound edges covered with surgical towels. . .towels were left in situ for the duration of the entire surgery and were removed immediately before closure of the fascia."	1.) randomization stratified by center 2.) trial arms were not significantly different with respect to: age, gender, BMI, diabetes mellitus, previous chemotherapy, previous radiotherapy, smoking status, alcohol consumption 3.) trial arms were not significantly different with respect to: type of surgery, skin prep used, duration of surgery, NNIS risk score, degree of wound contamination, antibiotic prophylaxis or treatment 4.) risk-factor adjusted analysis of SSI incidence (as sensitivity analysis)
Nyström & Bröte ^47^	1980	Sweden	Open appendectomy	• Preoperative clinical diagnosis of acute appendicitis	• Children below 10 years;	1	single-ring	"Vi-drape (Park-Davis) having a ring diameter of five inches (12cm) was used throughout the investigation"; "If the patient was allocated to the treatment group the drape was now (after McBurney incision) placed in position."	"Without plastic wound drape"	1.) all operation fields were prepared with 0,5% chlorhexidine in 70% alcohol 2.) all wounds were closed for healing by first intention 3.) drains were not used in all patients 4.) antibiotics were instituted at operation in cases with obvious or suspected gross contamination as decided by the surgeon (14 treatment; 18 control) 5.) comparable with resepect to: age, gender, degree of appendicitis,
Nyström, Broomé et al. ^30^	1984	Sweden	Colorectal surgery	• Adults admitted for elective colorectal surgery involving opening of the bowel	• None specified	2	single-ring	"With wound ring drape"; "The drape is made of a polyvinyl plastic sheet with a central hole which is fitted with a plastic frame that can be adjusted to match the size of the incision (Op-drape, Triplus)"; "The wound ring drape was adjusted to appropriate size and inserted into the abdomen before opening of the bowel."; "In patients having an ileostomy or colostomy made, the drape usually had to be removed before this final phase of the operation."	"Without wound ring drape"	1.) mechanical cleansing of the bowel was standardized for all patients 2.) all patients received antibiotic prophylaxis 3.) randomisation was stratified to antibiotic regime 4.) all wounds were closed 5.) operations were performed by senior surgeons or by registrars under supervision and were distributed evenly among surgeons 6.) trial arms were comparable with respect to: age, diagnoses, operative procedure
Pinkney et al. ^48^	2013	UK	Abdominal surgery	• Patients over 18 of age • Undergoing laparotomy for any surgical indication through a major incision in both elective and emergent settings	• Laparoscopic and laparoscopic assisted procedures • Previous laparotomy within 3 months • Patients unable to give informed consent	21	single-ring	3M Steri-Drape Wound Edge Protector during intra-abdominal part of the operation + standard intraoperative care as by local hospital policy	Standard intraoperative care as by local hospital policy without wound edge protector. With or without towels.	1.) stratified randomisation according to urgency, likelihood of opening the viscus, likelihood of creating a stoma 2.) trial arms were comparable with respect to: age, gender, BMI, diabetes mellitus, smoking status, malignancy, urgency of operation, ASA grade, operation characterisitics, degree of contamination, skin prep used, NNIS risk index, duration of surgery, prophylactic antibiotic given
Psaila et al. ^28^	1977	UK	Abdominal surgery	• Abdominal surgery	• Patients receiving preoperative antibiotics (with the exception of non-absorbable sulphonamides used for bowel preparation) were not included in the trial.	1–2*	single-ring	1.) Ring drape: "Vi-Drape (Parke, Davis & Co.) was the plastic ring wound drape tested. This was placed through the wound itself, the ring being permitted to expand against the inner aspect of the abdominal wall and the drape being being drawn over the wound surfaces" in addition to standard linen towels. 2.) Adhesive drape: "The adhesive skin drape used was Steri-Drape (Minnesota Mining and Manufacturing & Co.); this was applied to the skin at the operative site over the linen towels" in addition to standard linen towels	"Linen towels alone were used"	1.) standardized skin prep for all patients 2.) "a standard two-layer method of wound closure, using continuous chromic catgut and monofilament nylon, was employed in the majority of cases" 3.) patients receiving peroperative antibiotics (with the exception of non-absorbable sulphonamides used for bowel preparation) were not included in the trial. 4.) comparable type of surgery; degree of contamination, gender
Redmond et al. ^49^	1994	Ireland	Gastrointestinal surgery	• All laparotomies in which the wound was classified as clean-contaminated, contaminated or dirty. • All patients undergoing gastrointestinal surgery were eligible.	• None specified	1	single-ring	"Wound-edge protector"	"No protection"	1.) Antibiotic prophylaxis and skin preparation were standardized 2.) Study groups were matched for age, sex, anaesthesia, skin preparation and operation time.
Reid et al. ^25^	2010	Australia	Colorectal surgery	• Older than 18 years of age • Scheduled for an elective open colorectal resection • All patients received colorectal anastomosis	• Patients who were cognitively impaired or otherwise unable to give informed consent were excluded from the study. • Patients undergoing laparoscopic colorectal resection were also excluded because of the concern of extraction site metastases in the abscence of wound protection.	4	double-ring	Alexis wound protector (Applied Medical, Rancho Santa Margarita, CA, USA) was used. "Patients in the intervention group had the wound protector placed once the peritoneum was open and adhesions to the anterior abominal wall were cleared. Treating surgeons used extra retraction where required by the retractors of their choice."	"In the control group wound retraction was achieved by retractors routinely used by the treating surgeon. "	1.) all patients received a single dose of i.v. prophylactic antibiotics according to Australian Therapeutic guidelines 2.) all patients had skin preparation with betadine 3.) ventilation was maintained intraoperatively with 80% oxgen an no nitrous oxide was used. Oxygen was alsocontinuously administered for 24 hours postoperatively 4.) Patient warming devices were used intraoperatively and in the recovery ward; 5.) wound closure was standardized in all patients 6.) mobilization and diet was standardized for all patients 7.) trial arms were comparable with respect to: age, gender, BMI, rate of mechanical bowel preparation, immunosuppression, preoperative chemoradiotherapy, diabetes, anemia, malnutrition, alcohol abuse, smoking status, skin disease, hypertension, ASA score, cancer diagnosis 8.) trial arms were comparable with respect to: type of operation, stoma creation, duration of operation, blood loss, hospital type, preoperative blood transfusion, usage of drains.
Sookhai et al. ^50^	1999	Ireland	Abdominal surgery	• Transabdominal surgery for gastrointestinal disease	• None specified	1–2*	single-ring	"Impervious wound edge protector"	"No wound-edge protector"	1.) all patients received a standard systemic antibiotic prophylaxis 2.) all patients received povidone-iodine skin preparation 3.) groups were matched in regard to smoking status, pre-operative hospital stay, mean operation time, intraoperative temperature, number of blood units transfused.
Theodoridis et al. ^51^	2011	Greece	Cesarian section	• Pregnant women who underwent cesarian section, elective or emergent	• Suspected chorioamnionitis	1	double-ring	"Alexis wound retractor was placed, with one retraction ring being inserted into the peritoneal cavity."	Conventional Doyen retractor	1.) trial arms were comparable for indications, age, BMI, gestational diabetes,insulin therapy, mean gestational week, median birth weight 2.) Standardized operation procedure 3.) all patients received povidone-iodine skin preparation 4.) all patients received cefuroxim sodium as standard prophylactic antibiotic therapy.
Alexander-Williams et al. ^52^	1972	UK	Gastrointestinal operations	• Patients were chosen for the trial who were to have a midline or paramedian laparotomy associated with the opening of some part of the bowel or biliary tract.	• None specified	1	single-ring	"Vi-Drape wound protectors (Park-Davis) were used during the operations"	"Either no wound protection or standard permeable cloth wound guards"	1.) "Apart from the use or non-use of the Vi-Drape abdominal wound protector, there was no other variation from the usual operative routine"; 2.) Groups were comparable with respect to: gender, median age, antibiotic prophylaxis and treatment;

NR: not recorded. * unclear whether trial was single center or performed at two trial sites.

**Table 2:** Endpoints, surgical site infections and outcomes of included studies. NR: not recorded.

Study	Outcomes	SSI definition	Duration of follow up	Number of patients intervention group	Number of patients control group	SSI rate intervention group	SSI rate control group	number of patients excluded after randomisation (group)
Baier et al.^39^	SSI	1.) "The wounds were examined. . . according to the CDC definition of SSI". 2.) Unclear if only superficial/deep SSI were assed or superficial/deep/organ space infections	1.) Daily till discharge. 2.) Phone contact on postoperative day 30	98	101	20	30	n = 33 • intervention: n = 16 reoperation, n = 1 withdrawn consent • control: n = 16 reoperation
Bätz et al. ^40^	SSI, microbiological results	"Disruption of wound healing was defined as any spontaneous or surgical reopening of the abdominal wound with pus discharge"	• Regular follow-up visits; • Duration unclear	25	25	1	7	NR
Cheng et al. ^41^	SSI, postoperative pain	"SSIs were diagnosed according to the Centers for Disease Control and Prevention (CDC) criteria". Unclear if only superficial SSIs or all SSIs were recorded	• "Incisions were inspected on a daily basis from the second postoperative day." • Clinical follow-up on postoperative day 30	34	30	0	6	n = 8 (group not reported)
Gamble & Hopton ^42^	SSI, microbiological results	"A wound was recorded as infected if a discharge occured from it."	• "Wounds were inspected on the third and eighth day and at the outpatient department within one month of discharge."	27	29	10	8	NR
Horiuchi et al. ^26^	SSI	SSI frequency and properties were analyed according to the criteria of the United States Centers for Disease Control and Prevention (CDC).	• Unclear	111	110	8	16	NR
Lee et al. ^43^	SSI	(Wound infection) "was defined as any significant SSI necessitating wound opening or treatment with antibiotics."	• "Patients were followed up for up to 3 weeks after operation."	61	48	1	7	n = 4 (group not reported)
Mihaljevic et al. ^45,46^	SSI, core body temperature	Primary endpoint of this superiority trial was the incidence of SSIs (superficial, deep, organ-space) according to the definition of the Centers for Disease Control and Prevention (CDC)	• Within 30 days after the operation (according to CDC)	300	294	27	52	n = 14 • intervention: n = 11 no laparotomy • control: n = 3 no laparotomy
Nyström & Bröte ^47^	SSI, microbiological results	"Only wounds with definite accumulation of pus requiring opening or which emptied spontaneously were recorded as being infected"	• Unclear. • "All patients were followed up postoperatively at the outpatients clinic or by answering a questionnaire sent by mail."	132	143	10	13	n = 14 • intervention: n = 11 no reason given • control: n = 3 no reason given
Nyström, Broomé et al. ^30^	SSI, bacteriological contamination	"Wound sepsis was defined as pus emptying spontaneously or upon incision"; "Since the drape protects the abdominal incision wound only, infectious complications from other sites of the operative field are not reported"	• Up to 30 days after the operation. • Detailed daily records of the postoperative course were kept.	70	70	7	6	NR
Pinkney et al. ^48^	SSI, Quality of life, Length of hospital stay, Cost effectiveness, Clinical efficacy of the device in relationship to the degree of contamination	Superficial SSI. . .based on the criteria developed by the Centers for Disease Control and Prevention (CDC)	• Within 30 days after the operation (according to CDC)	369	366	91	93	n = 25 • intervention: n = 7 missing data, n = 6 no laparotomy • control: n = 7 missing data, n = 5 no laparotomy
Psaila et al. ^28^	SSI, bacteriological contamination	"At least one of the following criteria was used to identify the presence of infection: 1. Erythema around the sutures or along the wound edge with an accompanying pyrexia, 2. Discharge of exudate or pus from the wound, 3. wound breakdown."	• Unclear. • "Wounds were inspected daily after the third postoperative day"	adhesive drape: 51 ring drape: 46	47	ring drape: 8; adhesive drape: 8	10	NR
Redmond et al. ^49^	SSI	"Wounds were deemed infected when there was overt pus or a culture-positive discharge."	• Wounds were assessed on days 5, 10 and 30 after operation	102	111	11	27	NR
Reid et al. ^25^	SSI, Experience of the surgeon with the device	"Primary outcome was superficial and deep SSI occuring within 30 days of surgery, as defined by the Centers for Disease Control and Prevention (CDC)."	• Within 30 days after the operation (according to CDC).	64	66	3 (superficial and deep)	15 (superficial and deep)	n = 5 • intervention: n = 2 protocol violation, n = 1 death • control: n = 1 protocol violation, n = 1 death
Sookhai et al. ^50^	SSI	"Postoperative wound infection was defined as the presence of a purulent discharge, a culture-positive wound discharge, pain/tenderness, localised swelling, erytema or cellulitis"	• Within 30 days of surgery	170	182	23	54	NR
Theodoridis et al. ^51^	SSI, Feasibility of device	"Wound infection, defined as wound dehiscence, pain or tenderness at the lower abdomen, localized swelling, redness, and heat or purulent discharge from the wound."	• Unclear. • "During postoperative hospitalisation."	115	116	0	3	NR
Alexander-Williams et al. ^52^	SSI	Mild wound infection (erythema), Moderate wound infection (exudate), Severe wound infection (pus)	• On day 3 and day 7 postoperative for the first 96 patients. • On day 7 and day 10 for rest of patients. In those patients discharged before the tenth day it was considered that there was no infection if the wound had been normal on the seventh day and if no subsequent wound infection was reported by the patient when he returned to follow-up	84	83	9 (+n = 1 wound infection occured at drainage site, not laparotomy site)	10	n = 3 (group not reported)

### Risk of bias

**Fig 3:**
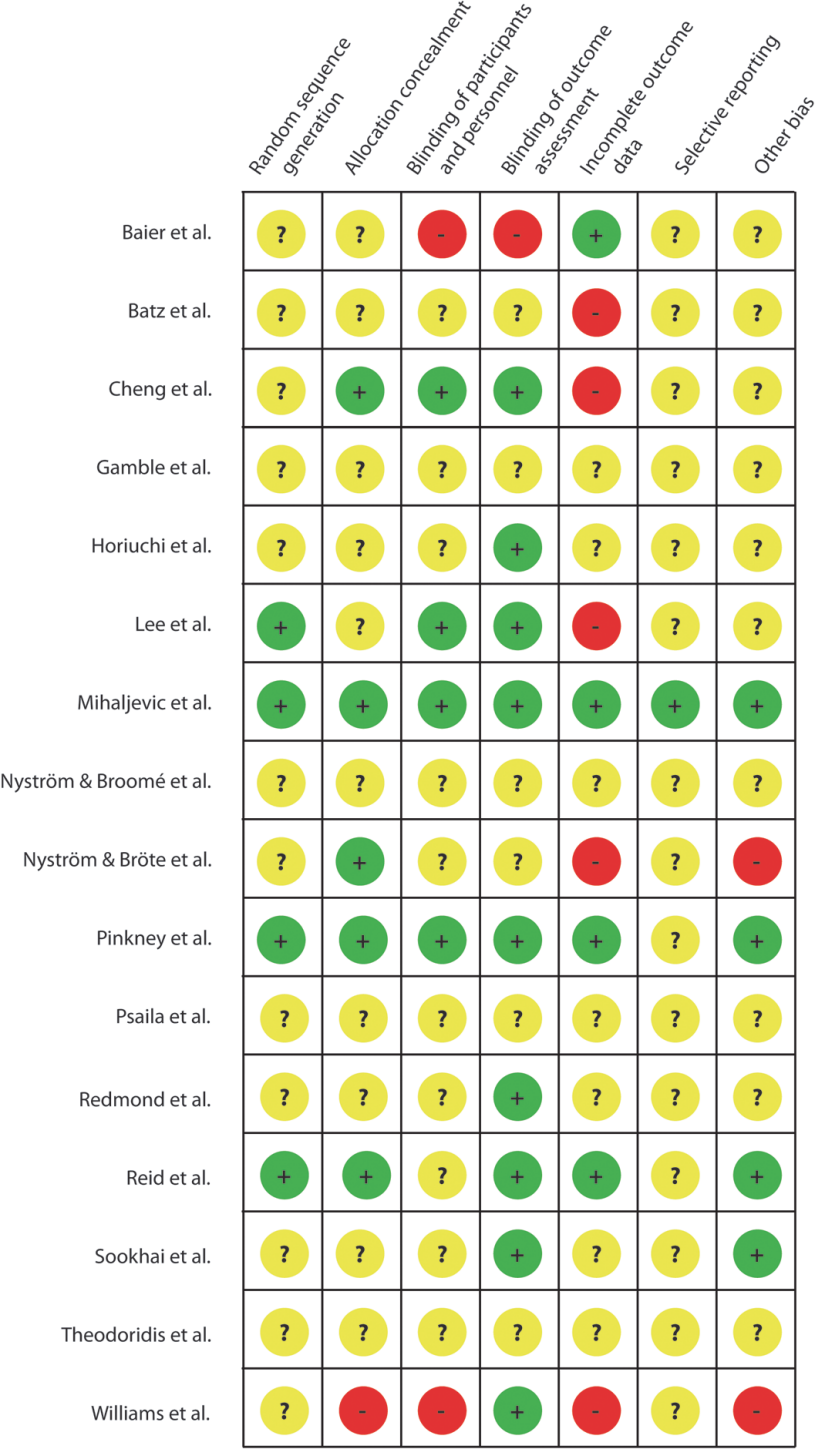
Risk of bias summary figure. Trials were rated in eight categories. + indicates low-risk of bias; − indicates high-risk of bias;? indicates unclear risk of bias.

Risk of bias is summarized in Fig 3 according to the Cochrane Collaboration proposal [37]. A risk of bias graph and a detailed assessment of bias for each individual study are provided in S2 Fig and S2 Table. Frequently, there was no report whether patients were excluded post randomization [27–29,31,43,50,52] or missing data of patients excluded post-randomization were not specified (attrition bias; Table 2) [41,42,44,48,53]. Many trials did not report if outcome assessors were blinded [29,31,41,43,48,52] or used an unblinded outcome assessment [40]. Only 4 trials described an adequate process of random sequence generation [26,44,46,49]. Similarly, few trials reported allocation concealment [26,42,46,48,49]. In the trial by Alexander-Williams, study personnel apparently had access to the patient allocation scheme [53]. Other forms of bias concerned the SSI definition employed and the length of follow-up or the failure to report conflicts of interest or funding sources [27–29,31,40,41,43,48,50,52,54]. In the trial by Alexander-Williams an industrial sponsor assisted with the design of the trial and preparation of the randomization schedule [53]. Furthermore, a number of trials failed to report comparability of study arms with respect to SSI risk factors (see Table 1). Bätz et al failed to clearly define SSI in their trial [41]. Lee et al. conducted a post-hoc interim analysis, which lead to discontinuation of the trial [44].

**Fig 4:**
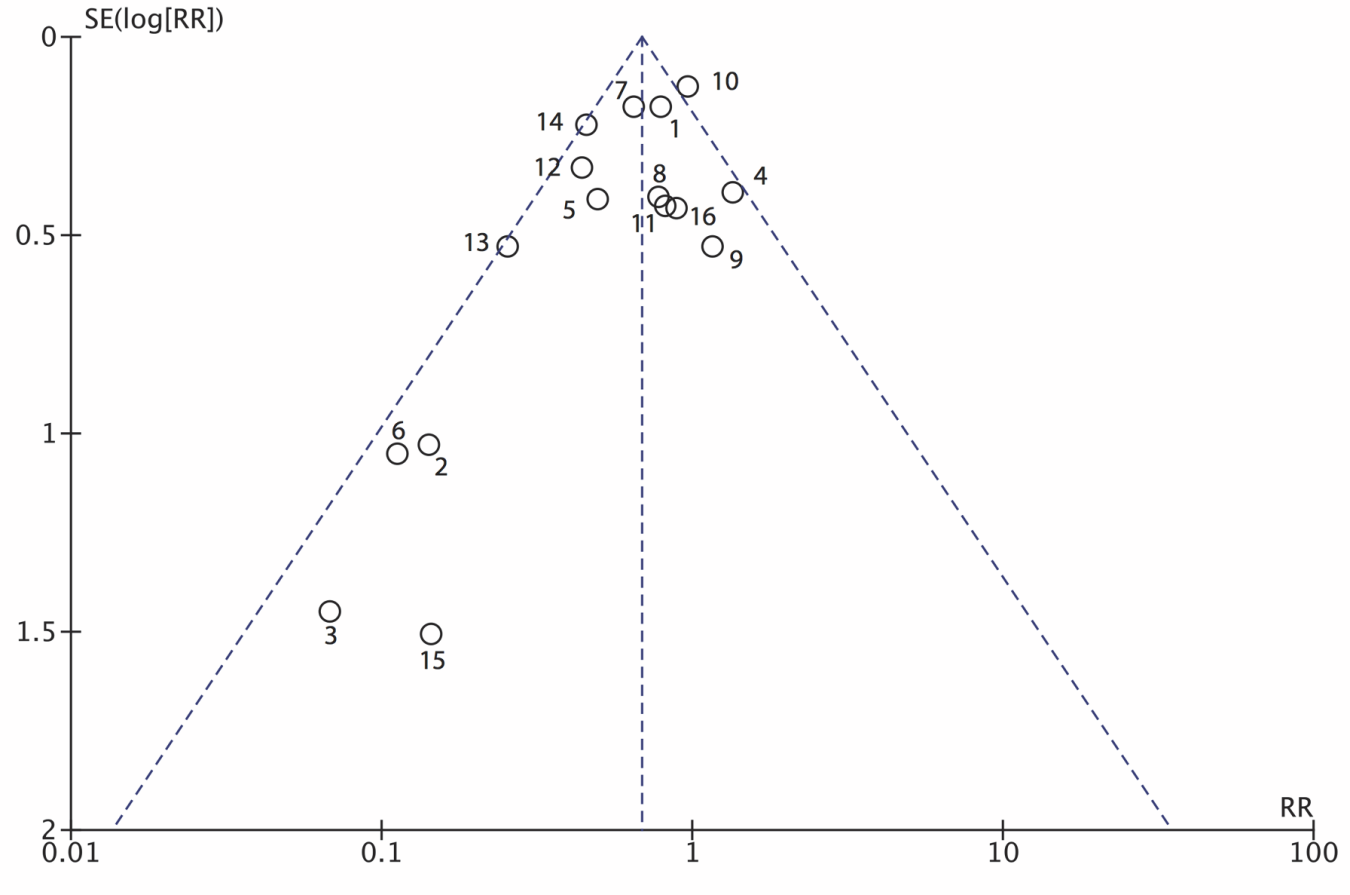
Funnel plot of the included RCTs comparing CWEPs with control (RevMan 5 output). 1: Baier et al; 2: Bätz et al; 3: Cheng et al.; 4: Gamble & Hopton; 5: Horiuchi et al.; 6: Lee et al.; 7: Mihaljevic et al.; 8: Nyström & Bröte; 9: Nyström, Broomé et al.; 10: Pinkney et al.; 11: Psaila et al.; 12: Redmond et al.; 13: Reid et al.; 14: Sookhai et al.; 15: Theodoridis et al., 16: Alexander-Williams et al.

The risk of publication bias was examined using a funnel plot (Fig 4). The asymmetry of the plot is due to 4 studies by Bätz et al. [41], Cheng et al. [42], Lee et al. [44], and Theodoridis et al. [52], that favor CWEPs but have a higher standard error of the effect estimate.

### Results of individual studies and synthesis of results

**Fig 5:**
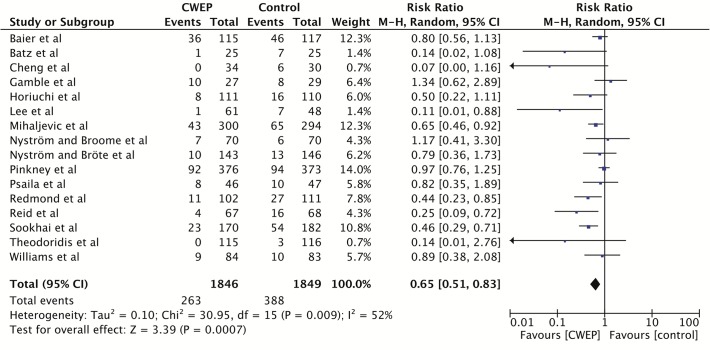
Individual trial data, pooled effect estimates and forest plot of the 16 randomized-controlled trials included in the meta-analysis. CWEPs vs. control with SSI as outcome parameter (RevMan 5.2 output).

The individual risk ratios and 95% confidence intervals (95%CI) for the random effects model meta-analysis of the 16 included RCTs are shown in Fig 5. The 6 trials published before 1990 all failed to show a statistically significant effect [29,31,41,43,48,53]. Of the 10 trials published after 1990, seven trials reported a significant reduction in SSIs in the CWEP group in their original report [26–28,42,44,46,50], a result which was not reproducible in our random effects model for the trials by Cheng et al. [42] and Horiuchi et al. [27] although both trials clearly favored CWEPs. Two trials after 1990 failed to show a significant reduction in SSI [40,49] and one trial did not elaborate its statistical analysis [52]. Cheng et al. reported the most favorable result for CWEPs (risk ratio, 0.07; 95%CI, 0.00–1.16) [42].

In the meta-analysis of all 3695 patients the application of CWEP significantly reduced the rate of SSI (risk ratio in the random-effects meta-analysis 0.65; 95%CI, 0.51–0.83; p = 0.009; I^2^ = 52%). When a fixed-effect model was used the pooled risk ratio was 0.68 (95%CI, 0.60–0.79; p<0.00001; I^2^ = 52%).

### Sensitivity analyses of pooled data

In order to test the robustness of our results, we performed three pre-specified sensitivity analyses. First, we counted all patients excluded post-randomization (i.e. with missing data) as SSI in both groups. In the resulting random-effects model meta-analyses the pooled risk ratio was 0.70 (95%CI, 0.54–0.89; p = 0.004; I^2^ = 55%) and 0.73 (95%CI, 0.64–0.83;p<0.0001;I^2^ = 55%) in a fixed-effects model in 3695 patients. These results indicate that in the unlikely event that all missing patients would have developed a SSI, CWEPs still conferred a significant benefit. Secondly, we analyzed a potential ‘worst case’ scenario for CWEPs by imputing all missing data as SSI in the CWEP group and as non-SSI cases in the control group in the same 3695 patients. In this highly unrealistic event the pooled risk ratio for SSI was 0.76 (95%CI, 0.58–1.02; p = 0.06; I^2^ = 63%) in a random effects model and 0.83 (95%CI, 0.72–0.95; p = 0.009; I^2^ = 63%) in a fixed-effects model. Finally, we conducted a meta-analysis excluding all trials that were at high overall risk of bias [40,44,52,53] (see methods section for definition and Fig 3). In this case the pooled risk ratio was 0.61 (95%CI, 0.45–0.83; p = 0.002; I^2^ = 59%) in a random-effects model and 0.67 (95%CI, 0.57–0.78; p<0.0001; I^2^ = 59%) in a fixed effects-model in 3065 patients.

Furthermore, CWEPs still conferred a significant advantage over controls if the three underpowered trials [41–43] were removed from analysis: pooled risk ratio 0.65 (95%CI, 0.51–0.83; p<0.001; I^2^ = 48%) in a random-effects model and 0.69 (95%CI, 0.60–0.80; p<0.001; I^2^ = 48%) in a fixed-effects model in 3525 patients. If only the 3 multicenter trials [26,46,47,49] with 1478 patients were analyzed a significant SSI reduction was found using a fixed-effects model (risk ratio 0.79; 95%CI, 0.64–0.96; p = 0.02; I^2^ = 76%), while the risk ratio was 0.67 (95%CI, 0.40–1.11; p = 0.12; I^2^ = 76%) in a random-effects model.

### Additional subgroup analyses

**Fig 6:**
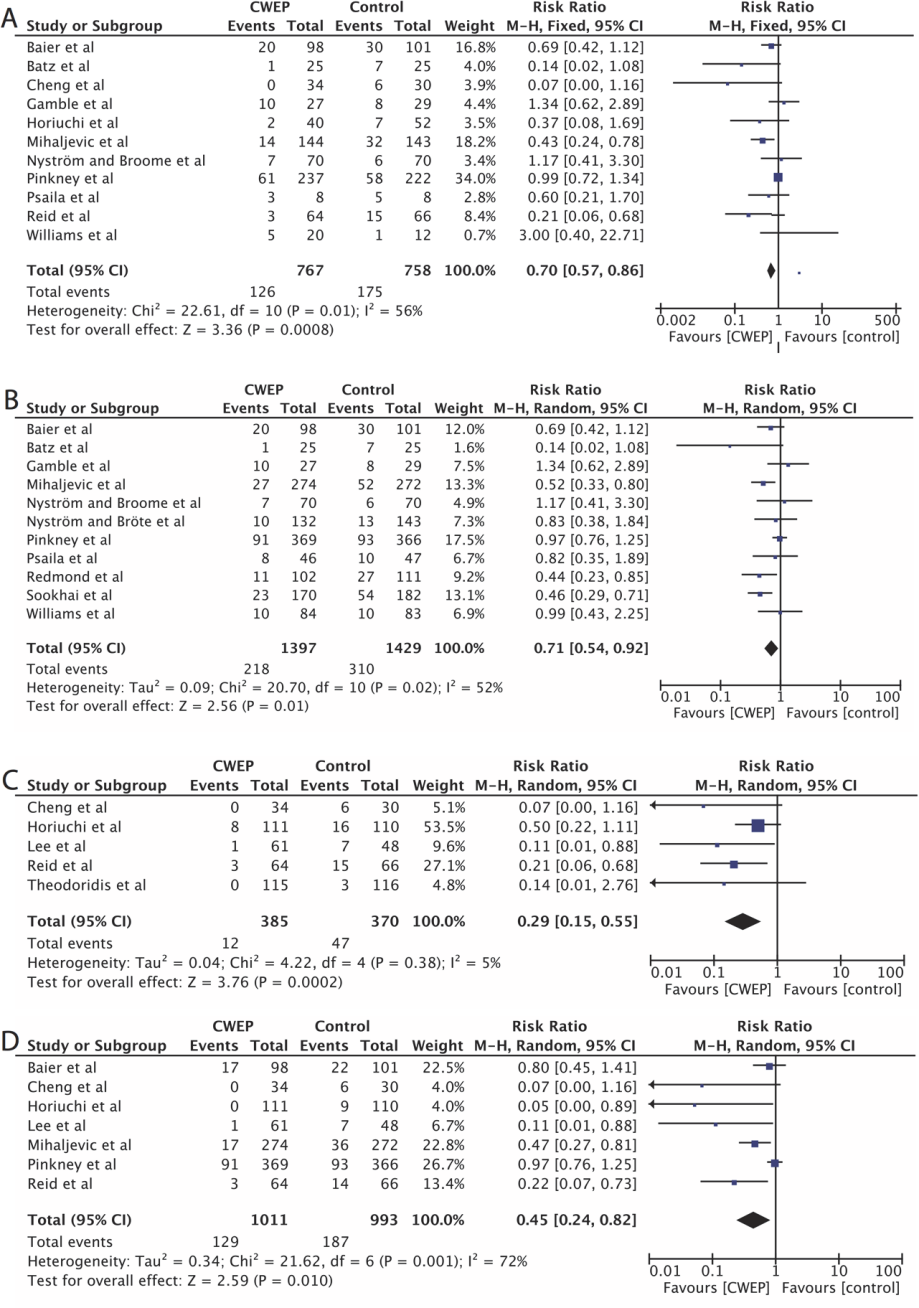
Prespecified subgroup analyses in the complete case population. A Individual and pooled data and effect sizes in colorectal surgery; CWEP vs. control. B Single-ring CWEPs vs. control. C Double-ring CWEPS vs. control. D Individual and pooled data and effect sizes in respect to superficial SSIs; CWEP vs. control.

A subgroup analysis of the effectiveness of CWEPS in patients undergoing colorectal surgery including 11 RCTs (reporting data of 1525 patients) [26,27,29,31,40–43,46,49,53] showed a pooled risk ratio 0.65 (95%CI, 0.44–0.97; p = 0.04; I^2^ = 56%) in the random-effects model and 0.70 (95%CI, 0.57–0.86; p = 0.0008; I^2^ = 56%) in the fixed-effects analysis, meaning that the effect size in the open colorectal surgery subgroup was comparable to the effect seen in general abdominal surgery (Fig 6A).

In a second set of subgroup analyses different types of CWEPs (single-ring or double-ring devices vs. control) were investigated. A total of 11 RCTs including 2862 patients were available for single-ring devices (Fig 6B). The pooled risk ratio in the random-effects meta-analysis was 0.71 (95%CI, 0.54–0.92; p = 0.01; I^2^ = 52%) and 0.72 (95%CI, 0.61–0.84; p<0.0001; I^2^ = 52%) in the fixed-effects model. Fewer trials were available on double-ring devices, as these models were introduced to the market much later. Five trials including 755 patients reported a statistically significant risk ratio of 0.29 (95%CI, 0.15–0.55; P = 0.0002; I^2^ = 5%) in the random-effect model and 0.26 (95%CI, 0.15–0.47; P<0.00001; I^2^ = 5%) in the fixed-effects model in favor of double-ring CWEP usage (Fig 6C).

In a next step we aimed to analyze the effect of CWEPs on different types of SSI depths (superficial, deep, organ-space) [17]. However, only two trials explicitly reported data on organ-space infections [26,27] (n = 17 cases) and five trials [26,27,40,42,44] on deep SSIs (n = 12 cases) making a meta-analysis infeasible. The pooled risk ratio for superficial SSIs in 7 RCTs [26,27,40,42,44,46,49] including 2007 patients was 0.45 (95%CI, 0.24–0.82; p = 0.01; I^2^ = 72%) in a random-effects model and 0.69 (95%CI, 0.56–0.84; p = 0.002; I^2^ = 72%) in a fixed-effects model.

**Fig 7:**
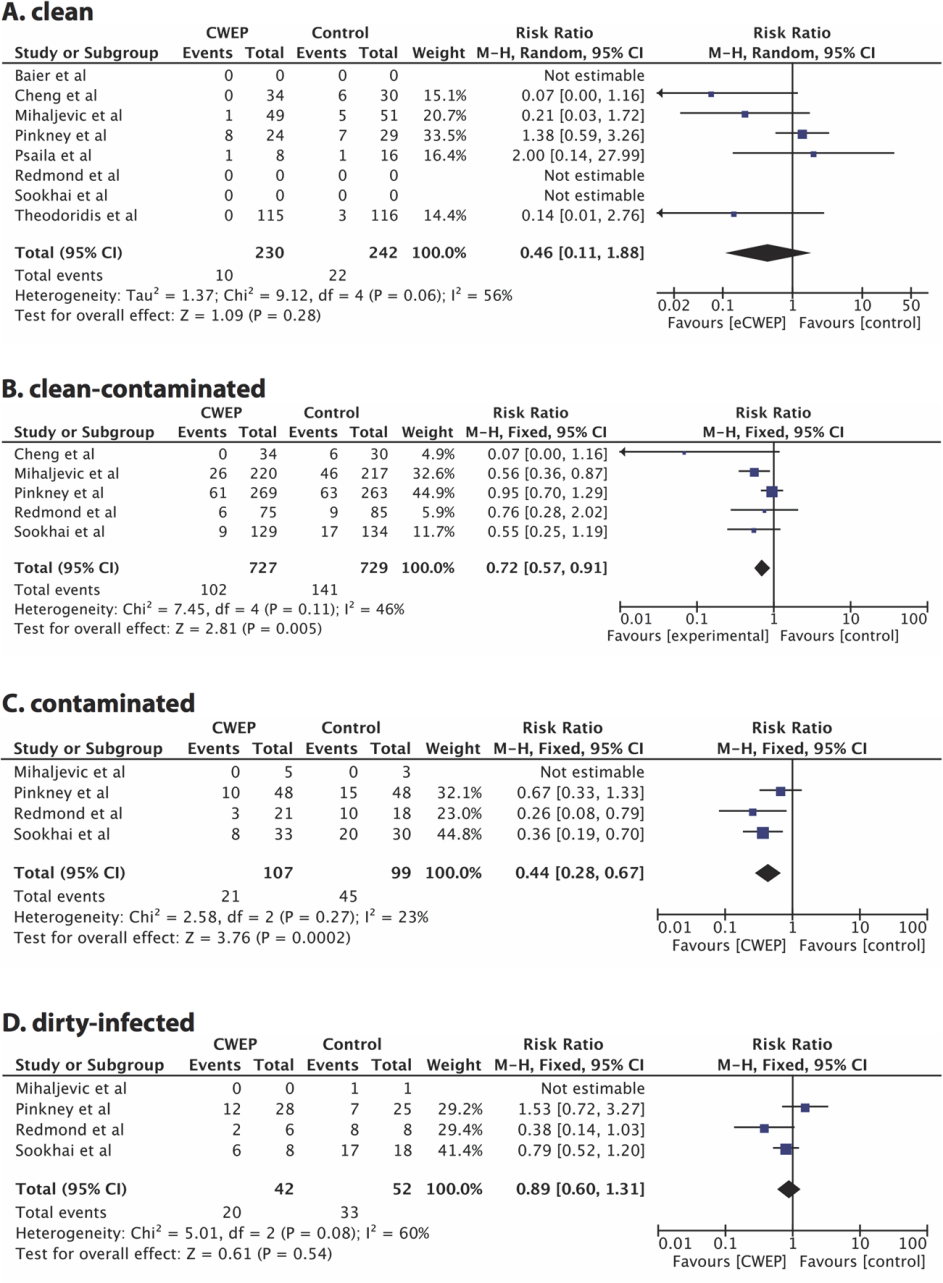
Exploratory subgroup analysis, CWEP vs. control in different degrees of contamination. A clean surgeries; B clean-contaminated surgeries; C contaminated surgeries; D dirty-infected surgeries.

Finally, we investigated the effectiveness of CWEPs vs. control in different levels of contamination (clean, clean-contaminated, contaminated, dirty-infected) (Fig 7A–7D). Five RCTs [29,42,46,49,52] including 472 patients reported data in clean operations, five trials [28,42,46,49,50] including 1456 patients in clean-contaminated surgery, four trials [28,46,49,50] with 206 patients in contaminated and the same four trials 94 patients in dirty-infected surgery. These subgroup analyses have to be treated with caution and are exploratory at best as patient and event numbers are small, and the description and definition of contamination grade varied between trials. The meta-analyses were conducted in a fixed-effects model as few trials were included [38]. The pooled risk ratios were 0.54 (95%CI, 0.29–1.03; p = 0.06; I^2^ = 56%) in clean surgeries (Fig 7A), 0.72 (95%CI, 0.57–0.91; p = 0.005; I^2^ = 46%) in clean-contaminated cases (Fig 7B), 0.44 (95%CI, 0.28–0.67; p = 0.002; I^2^ = 23%) in contaminated operations (Fig 7C) and 0.89 (95%CI, 0.60–1.31; p = 0.54; I^2^ = 60%) in dirty-infected cases (Fig 7D).

## Discussion

### Summary of findings

SSIs remain one of the most frequent complications in open abdominal surgery. CWEPs are a simple and fast intervention that hold promise to reduce SSIs, but reports on its effectiveness have yielded conflicting results requiring a systematic review of available data. This was confirmed by our literature search, in which we identified 9 RCTs that reported positive results for CWEPS [26–28,41,42,44,46,50,52] while 7 RCTs failed to show a benefit [29,31,40,43,48,49,53]. Furthermore, two previous systematic reviews based on limited patient numbers have uncovered the methodological flaws in many previous studies investigating CWEPS [32,33] and have called for high-quality multicenter RCTs to be performed. Several high-quality trials including more than 1800 additional patients have been published since, necessitating our current reevaluation and meta-analysis, which elucidates the clinical effectiveness of CWEPs based on sound evidence.

We calculated a pooled risk ratio for the 3695 included patients of 0.65 (95%CI, 0.51–0.83) in a random-effects model. When a fixed-effect model was used the pooled risk ratio did not differ greatly (risk ratio 0.68; 95%CI, 0.60–0.79) indicating moderate inter-study heterogeneity. This was confirmed by the I^2^ statistics (52%). Results indicate that CWEPs significantly reduce SSI in open abdominal surgery by 32–35% compared to controls. This would translate in a number-needed-to-treat of 15 (95%CI 10.9–23.3) i.e. 15 patients wound need to be treated with a CWEP to prevent one SSI. Given the large number of included RCTs and patients, these findings seem reliable. Furthermore, several of the included trials were conducted with high methodological standards and exhibited low-risk of bias [26,46,49] (Fig 3). In addition, we have included all randomized patients in our analyses as far as data were available and not just complete case data to prevent attrition bias as the proportion of missing outcome data in some trials compared with the observed event risk may well have induced clinically relevant bias.

Choosing a conservative way of imputation (deaths and relaparotomies were counted as SSIs) further supports our findings. Finally we conducted a number of sensitivity analysis to test the robustness of our results. Neither counting all patients excluded post-randomization as SSIs nor by excluding trials that were deemed at overall high risk of bias or underpowered trials changed the significance of our findings. By constructing a ‘worst case’ scenario (counting all missing data as SSI in the CWEP and as non-SSIs in the control group) however, showed a statistically significant result only in the fixed-effect model (RR 0.83; 95%CI, 0.72–0.95) but not for the random-effect model (RR 0.76; 95%CI, 0.58–1.02).

As CEWPs have been reported to be more effective in contaminated and colorectal operations, we have conducted several subgroup analyses. Pooling data from 11 RCTs and more than 1500 patients confirmed an overall significant effect of CWEPs in colorectal surgery, but the effect size (risk ratio 0.65; 95%CI, 0.44–0.97) was comparable to the one seen for all open abdominal patients. This is not surprising, if one assumes that most colorectal surgeries were clean-contaminated cases which were by far the most frequent type of surgeries conducted in the included RCTs (1456 cases out of 2228 operations that specified the degree of contamination). This might also explain that a similar risk ratio was found in the subgroup of clean-contaminated surgeries (risk ratio 0.63; 95%CI, 0.40–0.99). The subgroup analysis for clean, contaminated and dirty-infected surgeries have to be treated with caution and are exploratory at best, because of the limited number of patients, high CIs and high risk of bias of some of the included trials. However, our meta-analysis currently does not support the use of CWEPs in dirty surgeries. One might hypothesize that in dirty operations a contamination of the abdominal wall and subcutaneous tissue might occur upon laparotomy by spillage, i.e. before the CWEP can be put into place.

It was proposed that double-ring devices might be more effective than single-ring devices in protecting against SSIs [33]. Although we found the same phenomenon in our subgroup analysis (Fig 6B and 6C), as the pooled risk ratio for single ring devices was 0.71 (95%CI, 0.54–0.92), but 0.29 (95%CI, 0.15–0.55) for double-ring devices, we believe that this data does not sufficiently support the hypothesis. For single ring devices 11 RCTs with more than 2800 patients were available including two large multicenter trials with high methodological quality [46,49], while only 5 RCTs including 755 patients studied double-ring devices. With the exception of the study by Reid et al. [26] all of the double-ring studies exhibited considerable risk of bias [27,42,44,52]. Furthermore, three of these trials [42,44,52] were identified in the funnel plot analysis to exhibit considerable risk of publication bias (Fig 4).

### Limitations

There are several limitations of our analysis. First, although our analysis was confined to studies with clinical definitions of SSI, numerous different definitions of SSI were used in the included RCTs (Table 2). This is in line with a report by Bruce et al. who reported more than 40 different SSI definitions in 80 publications in a 5-year period [55]. Most importantly, a number of authors did either not specify the length of follow-up [27,29,41] or used inadequate follow-up durations [44,52,53]. We have tried to eliminate the bias of these studies by excluding these trials in our sensitivity analysis. Only 6 trials used the internationally accepted SSI definition of the CDC [26,27,40,42,46,49].

Secondly, not all trials used the same control intervention (Table 1). Some authors used surgical towels [29,40,42,46], no wound edge coverage [27,44,50], both [49,53], incise drapes [41] or did not clearly specify the coverage of wound edges in the control group [26,28,31,43,48,52].

Furthermore, a number of trials failed to report clear inclusion and exclusion criteria (Table 1). Therefore, the risk of SSIs cannot be accurately assessed in these patient populations. Although a number of risk factors for SSIs have been well documented in the literature, including age, obesity, smoking, diabetes mellitus, comorbidities, preoperative antibiotic prophylaxis and the duration of the surgical procedure [17], many of the RCTs that we have included in our meta-analysis neither reported these risk factors sufficiently nor adjusted for them in their analysis (Table 1). Not controlling for SSI risk factors appropriately makes it difficult to assess the real effect of CWEPs.

Finally, the methodological quality of some of the included trials was poor. A frequent problem was the inadequate reporting of randomization procedures, allocation concealment, control interventions, outcome definitions or the length of follow-up. Frequently, trials failed to report on patients excluded post-randomization or did not specify the group allocation of excluded patients. Moreover, some trials exhibited flaws that could have influenced the outcome, like the unblended wound assessment in the trial by Baier et al. [40] or the lack of allocation concealment in the trial by Alexander-Williams et al. [53].

It is noteworthy, though, that a number of trials including a recent methodologically well conducted multicenter trial from the UK [49] failed to show a benefit for CWEPs. It remains unclear why CWEPs in open abdominal surgery failed to show a benefit in these trials, but it might well be, that aspects not accounted for in the study conduct influenced the efficacy of CWEPs. Of the seven studies that reported negative results in their original publications, 4 were conducted in the UK [29,43,49,53] and two by the same team of authors in Sweden [31,48]. The remaining one was conducted in Germany and although the authors failed to show a significant benefit of CWEPs, results clearly favored the intervention group (20 SSIs in 98 cases vs. 30 SSIs in 101 cases) [40]. Positive results, on the other hand, have been reported from Australia [26], Germany [41,46], Ireland [28,50], Japan [27], Malaysia [42] and the USA [44]. Pinkney and colleagues [49] have recently pointed out that changing of gloves and instruments after removal of the CWEP and before closure of the fascia and skin would not be standard practice in the participating UK trial sites, while it is in other countries and hospitals. These aspects warrant further evaluation in future studies.

## Conclusion

Our meta-analysis conducted of 16 RCTs and over 3600 patients showed that CWEPs are effective in reducing SSIs in open abdominal surgery compared to standard care. Our results are based on solid evidence from multiple trials, some with high methodological quality, and were consistent across multiple sensitivity analyses.

## Supporting Information

S1 PRISMA checklist (DOC)

S1 Fig Circular wound edge protectors (CWEP). *A* single-ring device. *B* double-ring device.
(TIF)

S2 Fig Risk of bias graph. (TIF)

S1 Table Excluded full-text articles and reasons for exclusion. (DOCX)

S2 Table Risk of bias table. (TIF)
