# Supplementary figures and images for: Wound Edge Protectors in Open Abdominal Surgery to Reduce Surgical Site Infections: A Systematic Review and Meta-Analysis
Source: PLoS One. 2015 Mar 27;10(3):e0121187. doi: 10.1371/journal.pone.0121187 (PMC4376627; doi:10.1371/journal.pone.0121187)

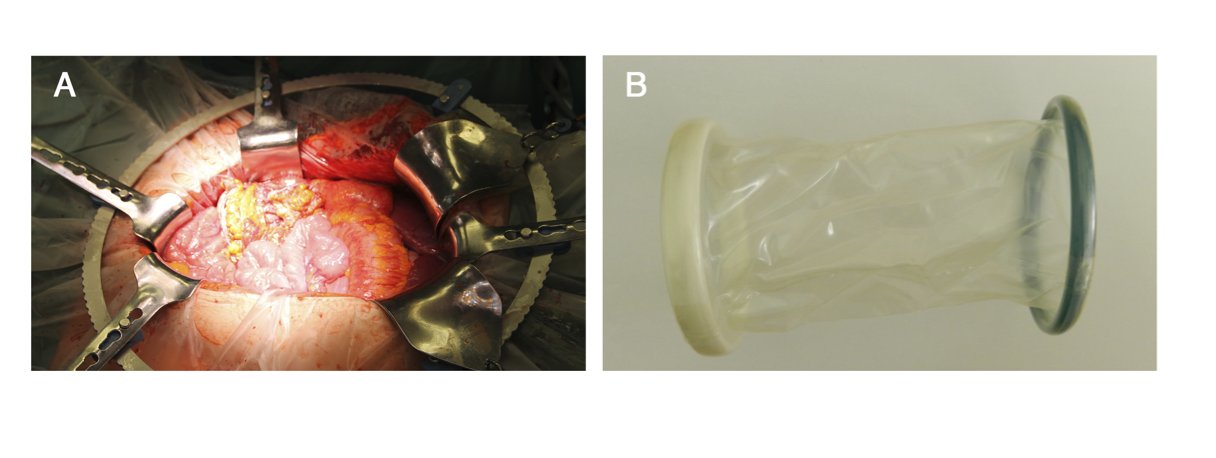

Supplement: S1 Fig — A single-ring device. B double-ring device. (TIF) [file pone.0121187.s002.tif]

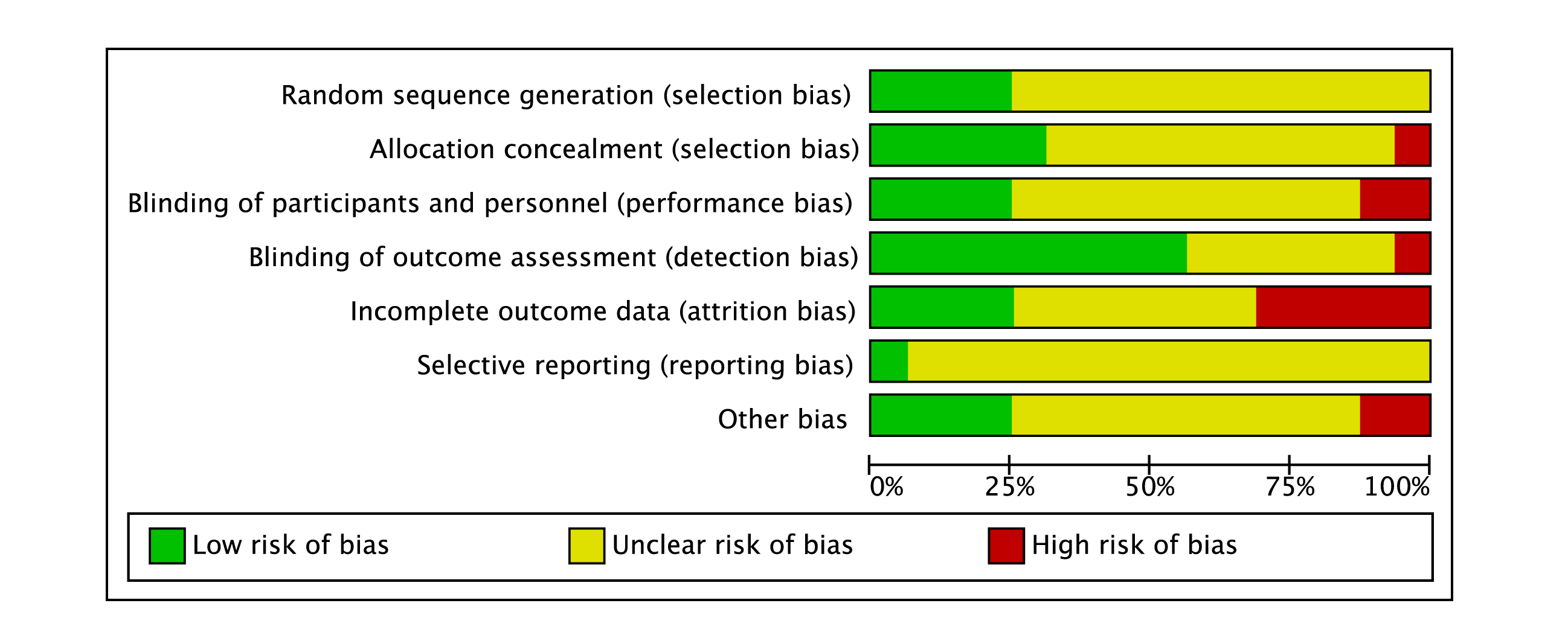

Supplement: S2 Fig — (TIF) [file pone.0121187.s003.tif]

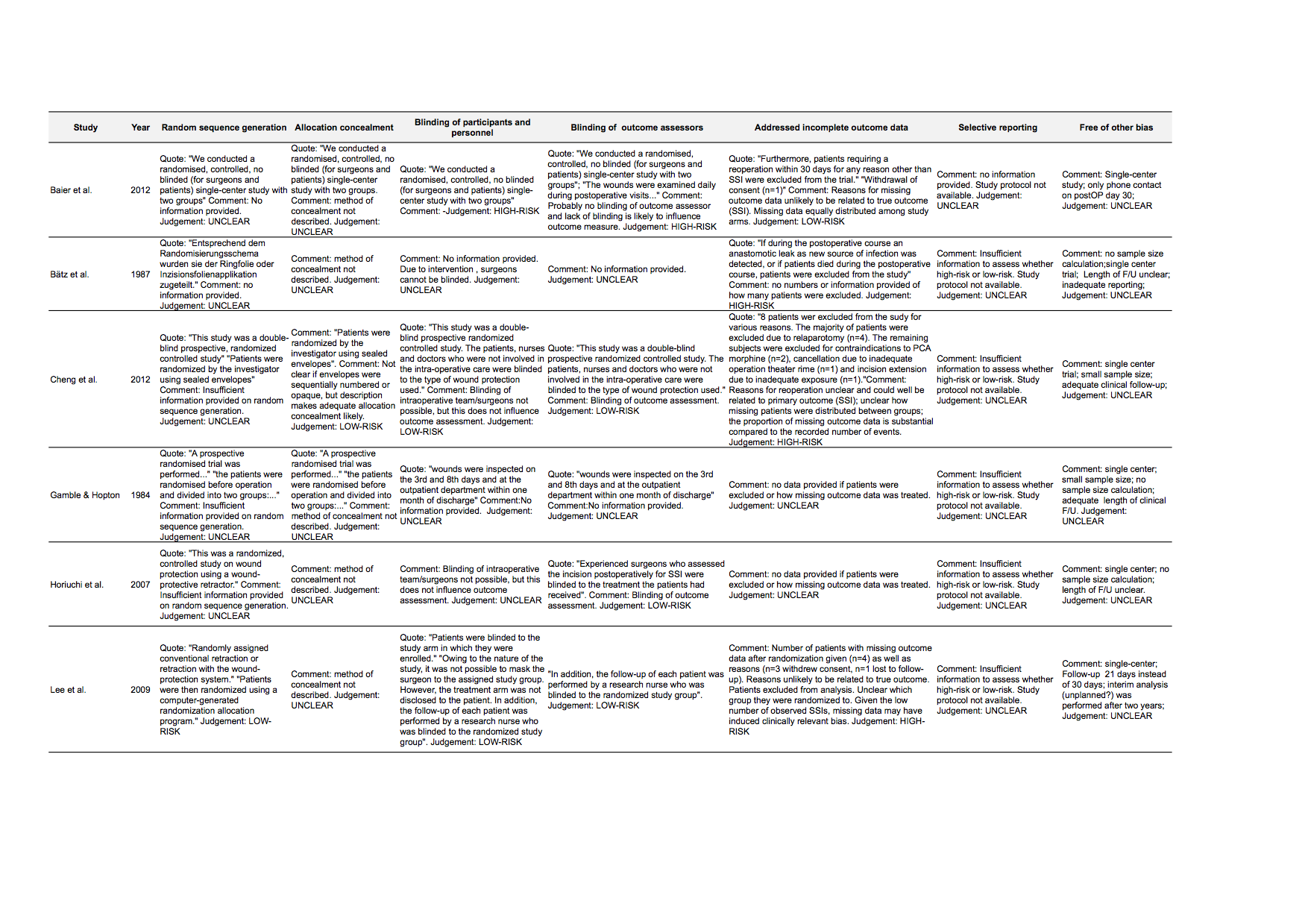

Supplement: S2 Table — (TIF) [file pone.0121187.s005.tif]
