# Supplementary material for: Wound Edge Protectors in Open Abdominal Surgery to Reduce Surgical Site Infections: A Systematic Review and Meta-Analysis
Source: PLoS One. 2015 Mar 27;10(3):e0121187. doi: 10.1371/journal.pone.0121187 (PMC4376627; doi:10.1371/journal.pone.0121187)
Supplement: S1 Table — (DOCX) [file pone.0121187.s004.docx]

Supplement 2

Excluded full-text articles and reasons for exclusion

|  | **Article** | **Reason for exclusion** |
| --- | --- | --- |
| **1** | Horiuchi T, Tanishima H, Tamagawa K, Sakaguchi S, Shono Y, Tsubakihara H, et al. A wound protector shields incision sites from bacterial invasion. Surg Infect (Larchmt). 2010 Dec;11(6):501–3. | Wrong outcome |
| **2** | Mohan HM, McDermott S, Fenelon L, Fearon NM, O’Connell PR, Oon SF, et al. Plastic wound retractors as bacteriological barriers in gastrointestinal surgery: a prospective multi-institutional trial. Journal of Hospital Infection. 2012 Jun;81(2):109–13. | Wrong outcome |
| **3** | Raahave D. Effect of plastic skin and wound drapes on the density of bacteria in operation wounds. Journal of Surgery. 1976 Jun;63(6):421–6. | Wrong outcome |
| **4** | Mohan H, McDermott S, Fenelon L, Fearron N, O’Connell R, Oon SF, et al. Retract and protect? Bacterial contamination of the incision site in abdominal surgery using a plastic wound retractor. A prospective multicentre study. Irish Journal of Medical Science, Springer, London Ltd., England 2011, p S272-272 | Wrong outcome |
| **5** | Mohan H, McDermott S, Fenelon L, Burke J, Oon S, O’Connell PR, et al. Prospective study of the bacteriological barrier effect of a plastic wound retractor in gastrointestinal surgery. 6th Scientific and Annual Meeting of the European Society of Coloproctology. September 2011, Copenhagen, Denmark. Colorectal Disease. 2011;13:11. | Wrong outcome |
| **6** | Fairclough J, Johnson D, Mackie I. The prevention of wound contamination by skin organisms by the pre-operative application of an iodophor impregnated plastic adhesive drape. J Int Med Res. 1986;14(2):105–9 | Wrong outcome |
| **7** | Pollock AV. Prevention of wound infection by an antiseptic wound protector. J R Soc Med. 1980 Nov;73(11):831. | Povidone iodone-soaked wound protector |
| **8** | Perrone G, Bianchi G, Pinardi L, Bocca M, Chiappa C, Colombo F, et al. Prophylaxis of surgical infections. Minerva Chirurgica. 1988;43(20):1721–8. | Bundle of interventions |
| **9** | Anthony T, Murray BW, Sum-Ping JT, Lenkovsky F, Vornik VD, Parker BJ, et al. Evaluating an evidence-based bundle for preventing surgical site infection: a randomized trial. Archives of Surgery. 2011 Mar;146(3):263–9. | Bundle of interventions |
